# Antioxidant and Antiproliferative Activities of Purslane Seed Oil

**DOI:** 10.4172/2167-1095.1000218

**Published:** 2016-04-25

**Authors:** Gai Guo, Li Yue, Shaoli Fan, Siqun Jing, Liang-Jun Yan

**Affiliations:** 1 College of Life Sciences and Technology, Xinjiang University, Shengli Road 14, Urumqi, Xinjiang 830046, China; 2 Department of Pharmaceutical Sciences, UNT System College of Pharmacy, University of North Texas Health Science Center at Fort Worth, 3500 Camp Bowie Boulevard, Fort Worth, TX 76107, USA

**Keywords:** *Portulaca oleracea* L. seed oil (PSO), Antioxidant, Anti-proliferation, Storage application

## Abstract

The aim of this study was to evaluate the antioxidant and antiproliferative activities of PSO *in vitro* and its application in horse oil storage. We determined the reducing power of PSO and its scavenging effects on hydroxyl (•OH) and 1,1-diphenyl-2-picrylhydrazyl radicals (DPPH•) and tested its stabilizing effects on horse oil storage. The results showed that PSO had remarkable, dose-dependent antioxidant activities, and it effectively prevented horse oil lipid oxidation. We treated cervical cancer HeLa cells, esophageal cancer Eca-109 cells and breast cancer MCF-7 cells with PSO using non-neoplastic monkey kidney Vero cells as controls. The results indicate that PSO significantly inhibited tumor cell growth in a time- and dose-dependent fashion. Our studies suggest that PSO may be used as a substitute for synthetic antioxidants in food preservation and may be potentially useful as a food and cosmetic ingredient. Meanwhile, the oxidative stress can cause hypertension, so PSO is expected to develop a health care products for the prevention and mitigation hypertensive symptoms.

## Introduction

Purslane (*Portulaca oleracea* L.) is a widely distributed weed that is extensively used not only as an edible plant but also as a traditional Chinese herbal medicine [1]. Both the leaves and seeds of purslane can be consumed orally or applied topically to soothe a skin allergy [2]. Many studies have demonstrated various pharmacological effects of this plant, such as antibacterial [3,4] hypoglycemic, [5] anti-hypoxia, [6] antioxidant effects, [7] antitumor activity, [8] and neuroprotective effects [9]. As a medicinal and edible wild plant, purslane is generally known as a “longevity food” due to its reputation as a “natural antibiotic”. Purslane contains many compounds, including flavonoids, [10] alkaloids, [11] omega-3 fatty acids, noradrenaline, alkaloids, coumarins, flavonoids, polysaccharides, and other active ingredients. In particular, purslane seeds are reportedly more effective in antioxidation than those from other herbs [12]. In previous studies, we have extracted purslane seed oil (PSO) with a 17.68% yield using an ultrasound-assisted enzyme hydrolysis combined with a Soxhlet extraction method. We then analyzed the fatty acid profile and content of the oil using a Gas Chromatography-Mass Spectrometer (GC-MS) [13]. Analysis of the PSO showed that alpha-linolenic acid reached 40.2570% followed by linoleic acid (29.4308%) and oleic acid (15.6103%). Saturated fatty acids represent 13.9455% of the total oil, while monounsaturated fatty acids and polyunsaturated fatty acids (PUFAs) account for 16.2877% and 69.6878%, respectively. Moreover, the linolenic acid content (40.2570%) in PSO is much higher than in camellia seed (0.27%), [14] grape seed (7.3%), [15] and olive (6.09%) oils [14] but is slightly lower than in flaxseed oil (41.22%) [12]. The content of linoleic acid (29.4308%) is much higher than in most other vegetable oils, such as flaxseed (15.44%), camellia seed (7.26%), grape seed (11.4%), and olive (0.56%) oils. It is well known that linolenic acid is an omega-3 fatty acid while linoleic acid is an omega-6 fatty acid, and both are the essential fatty acids that play important roles in human growth and development as well as in disease prevention [16]. Additionally, oils that are rich in omega-3 fatty acids are most likely beneficial to human health [17]. PSO is expected to show superior antioxidant activity and antitumor effects due to its high omega-3 fatty acid content. Thus, it is a good candidate as both a health food and a cosmetic ingredient.

However, there are few studies regarding the composition of fatty acids in purslane and PSO [13,18,19]. Moreover, to the best of our knowledge, there is no published research on PSO's antioxidant activity or antiproliferative effect on cancer cell lines. Therefore, in this study, we tested PSO's antioxidant activity on free radical scavenging and its inhibitory effects on tumor cell proliferation. Additionally, we also tested PSO as a preserving agent in horse oil storage to explore whether it can be used as a food-preserving agent.

## Materials and Methods

Fresh, mature purslane seeds were provided by Xinjiang Yuansen Agriculture Science and Technology Development Co., Ltd. PSO containing 40.2570% alpha-linolenic acid and 29.4308% linoleic acid was obtained by an ultrasound-assisted enzyme hydrolysis combined with a Soxhlet extraction method. The optimal preparation conditions of PSO were as follows: [13] for the hydrolysis process, 2% complex enzyme was used (the ratio of neutral protease to cellulase was 1:1), the liquid-solid ratio was 5:1, pH was 5.0, and the hydrolysis time was 2 h; for the sonication process, 40 W ultrasonic power was used, an ultrasonic bath temperature was set to 55°C, and the sonication time was 15 min. Petroleum ether was used as a solvent for the Soxhlet extraction. Fresh, untreated horse fat was purchased from a local market in Yili, Xinjiang, China. Liquid horse oil was obtained from the horse fat using a steam melting method followed by a refining process consistent with our previous studies [20]. Horse fat provides the raw material of which 31.08% of the lipids are unsaturated fatty acids, notably, palmitoleic acid (3.71%) and oleic acid (27.37%). The standard tertiary butylhydroquinone (TBHQ) that was used in vitro studies was purchased from Sigma Chemical Co. (St. Louis, MO, USA).

### *In vitro* antioxidant potential of PSO

TBHQ is a frequently used synthetic antioxidant; therefore, it was used as a reference material for evaluating the antioxidant activity of PSO in this study. The antioxidant activity of PSO was determined by various methods, such as 1,1-diphenyl-2-picryl hydrazyl (DPPH) radical scavenging, hydroxyl radical scavenging, and reducing power assays. PSO was dissolved in an anhydrous ethanol to form a series of final concentrations. Equal concentrations of TBHQ and PSO were used in each experiment. All tests were conducted in triplicate, and the mean values were plotted.

### Hydroxyl radical (•OH) scavenging activity assay

The phenanthroline-Fe2+ oxidation method previously described by Xiao et al. was used to measure hydroxyl radical scavenging activity [21]. Briefly, 4 mL of sodium phosphate buffer (pH 7.4) was added to a test tube and mixed with 1.5 mL of 5 mmol /L phenanthroline solution. Next, 1 mL of 7.5 mmol /L FeSO_4_ solution and 1 mL of a series of concentrations (0.5, 1, 2, 3, 4, 5, 6, 7 mg/mL) of PSO sample solution were added to the solution in sequence. Finally, 1.5 mL of double distilled water and 1.0 mL of 0.1% H_2_O_2_ were added. The absorbance of the final solutions was measured at 536 nm with a UV-visible spectrophotometer following incubation at 37 for 60 min. Deionized water and TBHQ were used as blank and positive controls, respectively. Antioxidant value was expressed as IC_50_, the concentration of the sample that caused 50% inhibition of hydroxyl radical formation.

### DPPH• radical scavenging activity assay

The scavenging activity of DPPH free radicals was measured according to the method reported by Ting et al, with slight modifications [22]. Briefly, 2 mL of 2×10^-4^ mol/mL DPPH was added to 2 mL series of concentrations (0.1, 0.3, 0.5, 1, 2, 3, 6, 10, 15, 20, 25, 30 mg/mL) of PSO in sequence. The reaction mixture was incubated for 30 min at room temperature in the dark, after which absorbance was measured at 517 nm using a spectrophotometer (TU-1900 PuXiTongYong, Beijing, China). Distilled water was used instead of the sample solutions as a control. TBHQ was used as a positive control. A mixture with an equal volume of distilled water and anhydrous ethanol was used as a blank control. The results were expressed as the amount of sample necessary to scavenge 50% of DPPH• radicals (IC_50_).

### Determination of reducing power

The reducing power of PSO was examined using the Prussian blue method [23]. Briefly, 1 mL of each PSO sample solution (0.01, 0.05, 0.1, 0.3, 0.5, 1.2, 2.4, 3.6, 4.8, 6, 7.2 mg/mL) was added to a solution containing 2.5 mL of phosphate buffer (pH 6.6) and 2.5 mL of 1% K_3_Fe(CN)_6_. The buffered solutions were then stored at 50 for 20 min, after which 2.5 mL of 10% trichloroacetic acid (TCA) was added. Next, 2.5 mL of distilled water and 2.5 mL of 0.1% FeCl_3_ were mixed with 2.5 mL of the previous mixtures. After 10 min, the absorbance value (A) was measured at 700 nm with a UV-visible spectrophotometer. The reference absorbance value (A_0_) was given by a blank reagent control. Deionized water was used as the blank control, and TBHQ was used as the positive control.

### Antiproliferative effect of PSO *in vitro*

HeLa, Eca-109, MCF-7, and Vero cells were obtained from the Xinjiang University Xinjiang Biological Resources Gene Engineering Key Laboratory (Urumqi, China). The in vitro antiproliferative activity of PSO was determined by measuring 3-(4,5-dimethylthiazol-2-yl)-2,5-diphenyltetrazolium bromide (MTT) dye absorbance in living cells (HeLa, Eca-109, and MCF-7) with Vero (normal) cell as controls. Briefly, cells were seeded in the 96-well, flat-bottomed plates containing 100 μL of a cell suspension with a known concentration per well and allowed to adhere at 37 in a humidified atmosphere containing 5% CO_2_. Usually, 5×10^4^ cells were seeded per well. PSO was dissolved in dimethyl sulfoxide (DMSO) and then filtered with filter membranes (0.45 μm and 0.22 μm) to achieve sterilization. 200 μL PSO at concentrations of 12.5 μg/mL, 50 μg/mL, 200 μg/mL, 800 μg/mL, 1600 μg/mL and 3200 μg/mL were added to their respective wells. In total, 20 μL of the MTT solution (5 mg/ml; Sigma-Aldrich, MO, USA) was then added at 24 h, 48 h, or 72 h for dyeing, and the cells were incubated for another 4 h at 37. After the incubation, the cell suspensions were centrifuged at 800 rpm for 10 min, and the supernatants were replaced by 200 μL DMSO to solubilize the formazan crystals formed in viable cells. Absorbance at 570 nm was measured using a microplate ELISA reader (Model 550, Bio-Rad, USA). The results were expressed as a percentage of control proliferation (100%). The IC_50_ value was expressed as the concentration of PSO that inhibited the growth of cells by 50% [8].

### Effect of PSO on the oxidative stability of horse oil during storage

The peroxide value (POV) is an indicator of lipid oxidation. The POV was determined by the Schall Oven method [24]. Briefly, an oil sample was incubated in a digital electric heating blast oven at 63 ± 1 constant temperature, and the POV values was measured once every 24 h, according to the National Standard of the People's Republic of China (GB/T 5538-2005/ISO3960:2001) [25]. The lower the POV, the stronger the oxidative stability of the sample. The POV (mmol/kg) of the sample was calculated by the following equation:

$$\text{POV}(\text{mmol}/\text{kg})=\frac{1000(V-V_{0})C}{2m},$$

where V is the volume of sodium thiosulfate (Na_2_S_2_O_3_) for the measurement (mL); V_0_ is the volume of Na_2_S_2_O_3_ for the blank test (mL); C is the concentration of Na_2_S_2_O_3_ solution (mol/L); and m is the weight of the sample (g).

To investigate the effect of PSO on horse oil storage stability, we added 0.5% (w/w) PSO to 30 g of horse oil. After thoroughly mixing the solution, the subsequent operations were performed according to the Schall Oven method. A blank test without added PSO and TBHQ was necessary. The TBHQ-added group was used as a positive control.

To investigate the effect of the dose of PSO on horse oil storage stability, we added PSO with different proportions of 0.05%, 0.25% and 0.5% to horse oil. After thoroughly mixing the solution, the subsequent operations were performed according to the Schall Oven method. The group without PSO added was regarded as a control.

### Statistical analysis

All results were expressed as the mean ± SD. The data were analyzed statistically using ANOVA. Statistical calculations were conducted using Graphpad Prism 5.0 (Graphpad Software Inc., San Diego, CA, USA). Values of p < 0.05 were considered significantly different.

## Results and Discussion

### Evaluation of PSO antioxidant activity in vitro

#### 

##### Hydroxyl free radical scavenging activity of PSO

Among the tested free radicals, hydroxyl free radicals are the most active and toxic. Thus, the hydroxyl free radical scavenging capacity can be used as an indicator of antioxidant activity. As shown in Figure 1a, the hydroxyl free radical scavenging capacity of PSO was enhanced at higher concentrations of PSO in a nearly linear relationship until a concentration of 4 mg/mL. The IC_50_ values of PSO and TBHQ were 1.388 ± 0.2033 mg/mL and 2.193 ± 0.1014 mg/mL, respectively. Moreover, at the same concentrations, the ranking of hydroxyl radical scavenging ability of PSO, TBHQ, almond oil, and grape seed oil was PSO > TBHQ> almond oil (IC_50_ 2.53 mg/mL) > grape seed oil (IC_50_ 6.66 mg/mL) [26]. Therefore, the hydroxyl free radical scavenging activity of PSO was the strongest among the tested samples. In the literature, grape seed oil is reported to contain linoleic (65.0%), linolenic (1.5%), oleic (17.0%), palmitic (8.0%), stearic (4.4%) and arachidonic (0.6%) acids; [27] while oleic (63-78%) and linoleic (12-27%) acids are the major fatty acids in almond oil [28]. It is clear that the predominant fatty acid in PSO is linolenic omega-3 fatty acid. Therefore, we reason that the stronger antioxidant activity of PSO may be attributed to its higher content of linolenic acid. Indeed, previous research indicates that an increased amount of omega-3 PUFAs may enhance antioxidant activity [29,30]. However, whether the observed effect is due to the omega-3 or to the other fatty acids in PSO needs to be further evaluated.

#### DPPH• free radical scavenging capacity of PSO

The DPPH• scavenging ability of PSO was enhanced when the oil concentration was increased (Figure 1b). A strong linear relationship is observed within the range of PSO concentrations from 3-20 mg/mL. Moreover, it is worth mentioning that PSO's antioxidant activity could only be detected at concentrations at or above 3 mg/mL. The IC_50_ values of DPPH• radicals scavenging of PSO and TBHQ were 11.16 ± 0.07075 mg/mL and 0.3783 ± 0.07886 mg/mL, respectively, and the result indicated that the DPPH• free radical scavenging capacity of PSO was weaker than that of TBHQ. Additionally, the DPPH• free radical scavenging capacity of PSO was higher than that of walnut oil (IC_50_ 147.0 mg/mL) [31] and weaker than that of flaxseed oil (IC_50_ 3.31 mg/mL) [32]. It has been reported that the major fatty acids in walnut oil are linoleic (60.42-65.77%), oleic (13.21-19.94%) and linolenic (7.61-13%) acids [31]. The main fatty acid components of flaxseed oil are alpha-linolenic (41.22%), linoleic (15.44%), and oleic (28.2%) acids. Obviously, the stronger DPPH• radical scavenging ability of PSO may be due to its higher content of linolenic omega-3 fatty acid.

##### Reducing power of PSO

Many studies have demonstrated that the activity of an antioxidant is closely related to its reducing power: the greater the reducing power, the stronger the antioxidant activity. Therefore, the reducing power can reflect the antioxidant activity [33]. The results in Figure 1c showed that the reducing power increased as the concentration of PSO increased, and there was a strong positive linear relationship. However, the reducing power of PSO was weaker than that of TBHQ.

In summary, we observed a strong antioxidant activity of PSO, suggesting that PSO is a likely candidate as a food and cosmetic ingredient.

### *In vitro* inhibitory effect of PSO on tumor cell proliferation

#### 

##### MTT assay for tumor cell proliferation inhibition

**Figure 2:**
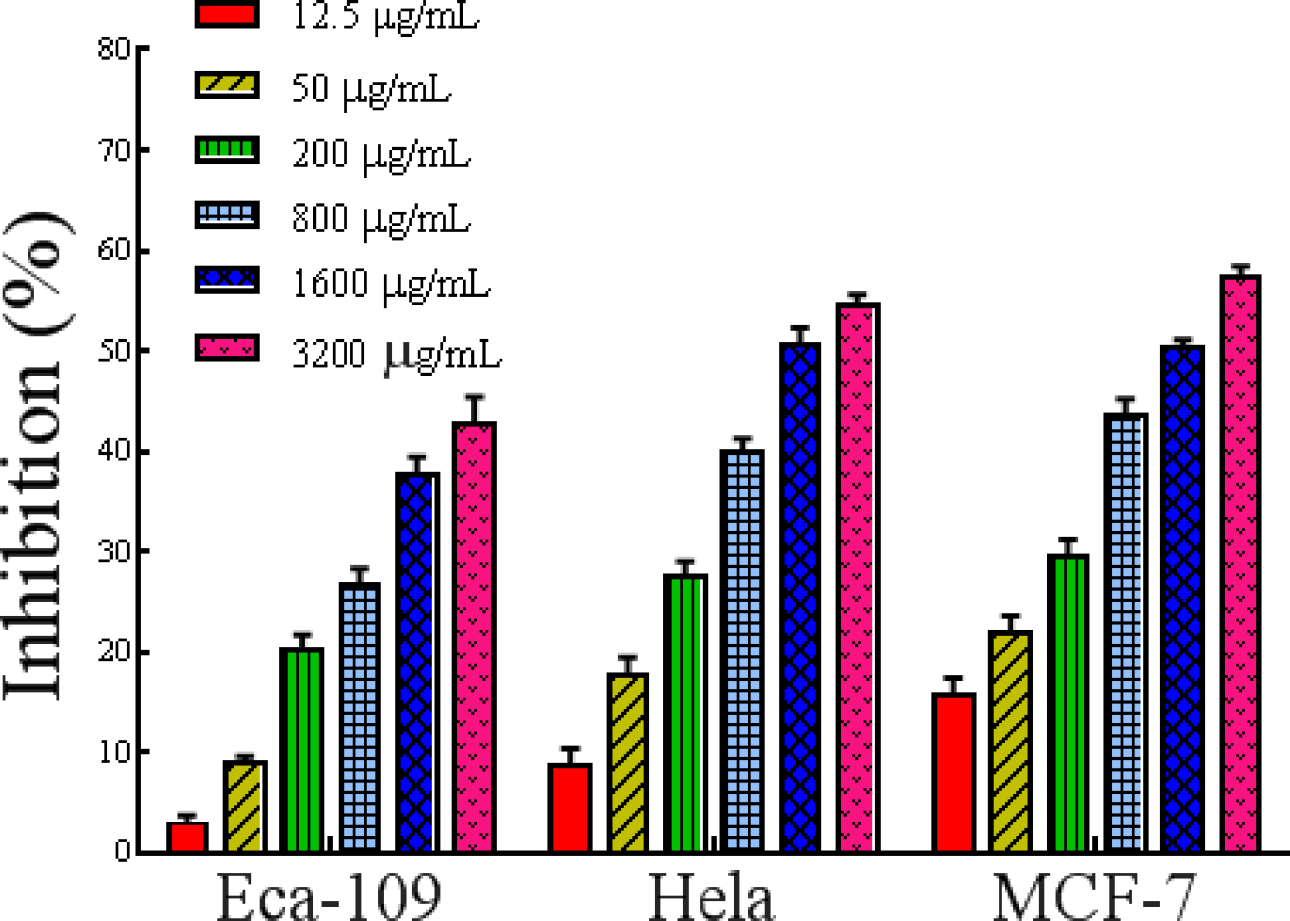
The curve of PSO inhibition of MCF-7, HeLa and Eca-109 cell proliferation for 48 h.

The antiproliferative effect of PSO on MCF-7, Eca-109 and HeLa cells was evaluated by an MTT assay, and the IC_50_ values were derived from the dose-response curves (Figure 2). PSO induced a significant dose- and time-dependent decrease in the proliferation rate of HeLa, MCF-7, and Eca-109 cells. The IC_50_ values of PSO against MCF-7, HeLa, and Eca-109 cells were 1566 ± 0.01691 μg/mL, 1844 ± 0.0217 μg/mL and 5366 ± 0.03851 μg/mL, respectively. PSO showed a stronger inhibitory effect on the proliferation of MCF-7 cells.

##### Growth inhibition of HeLa cells

**Figure 3:**
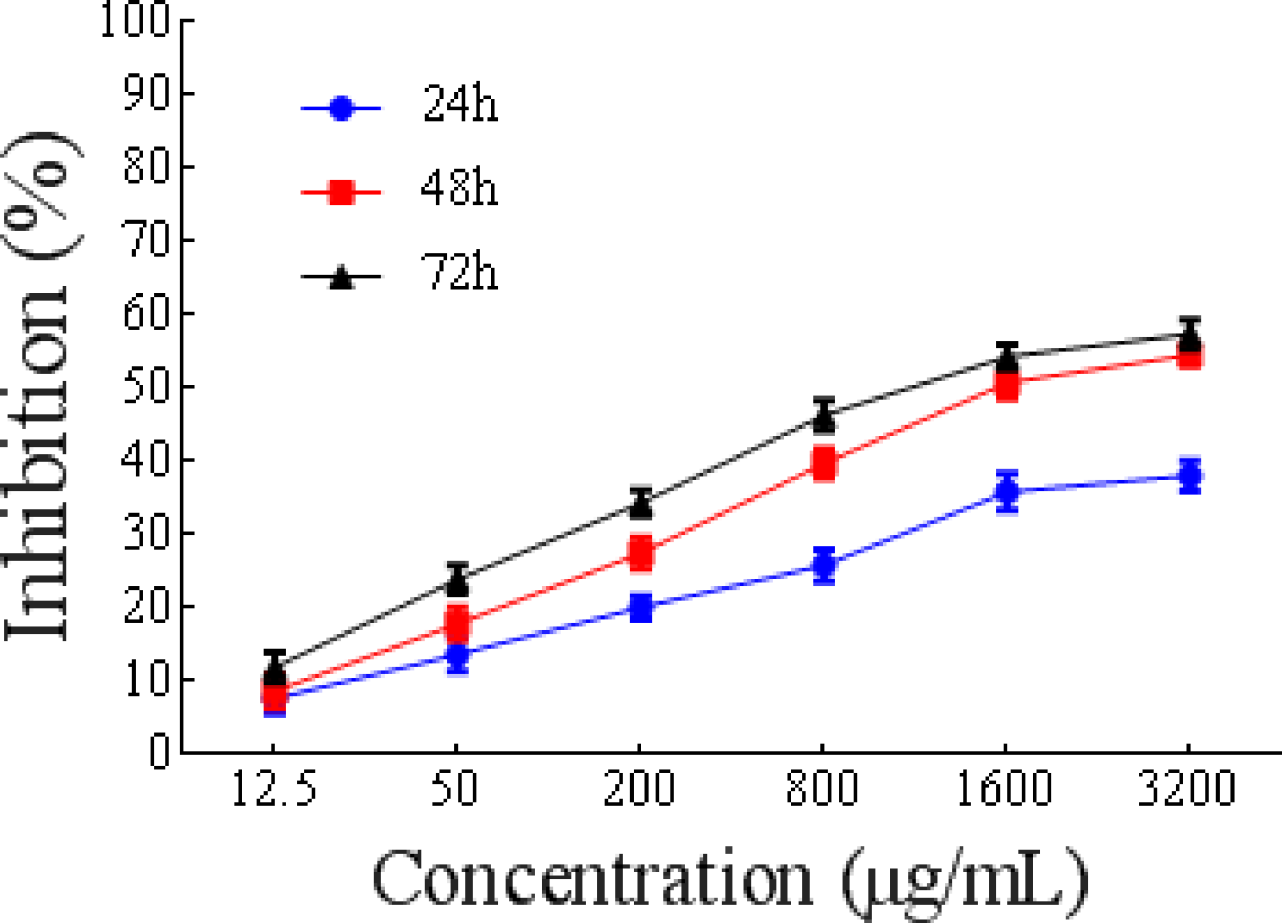
Time curve of HeLa cell proliferation as a function of different concentrations of PSO.

As shown in Figure 3, PSO inhibited the growth of HeLa cells in a dose- and time-dependent manner with IC_50_ values of 11634 ± 0.02706 μg/mL, 1844 ± 0.0217 μg/mL and 1179 ± 0.01989 μg/mL, respectively, after 24 h, 48 h, and 72 h. The same dual-dependent relationship was found by Cao et al. concerning essential oil from *Artemisia lavandulaefolia*, [34] Thus, PSO may significantly inhibit the proliferation of HeLa cells.

##### Effect on proliferation of Eca-109 cells

**Figure 4:**
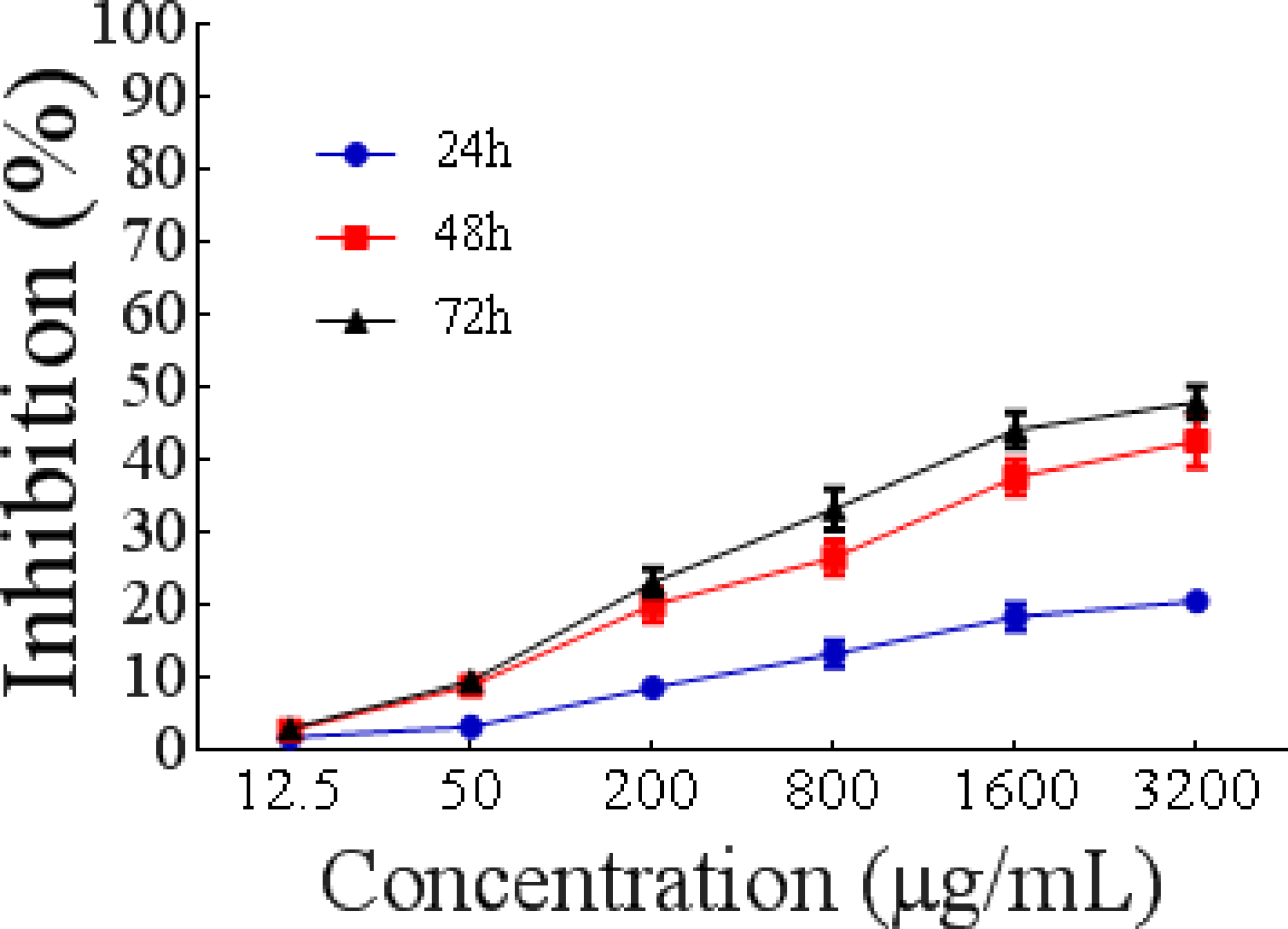
Time curve of Eca-109 cells proliferation as a function of different concentrations of PSO.

As shown in Figure 4, PSO inhibited the growth of Eca-109 cells in a dose- and time-dependent manner with IC50 values of 65540 ± 0.03675 μg/mL, 5366 ± 0.03851 μg/mL, and 3048 ± 0.03686 μg/mL, respectively, after 24 h, 48 h and 72 h. Therefore, PSO may significantly inhibit the proliferation of Eca-109 cells.

##### Effect on proliferation of MCF-7 cells

**Figure 5:**
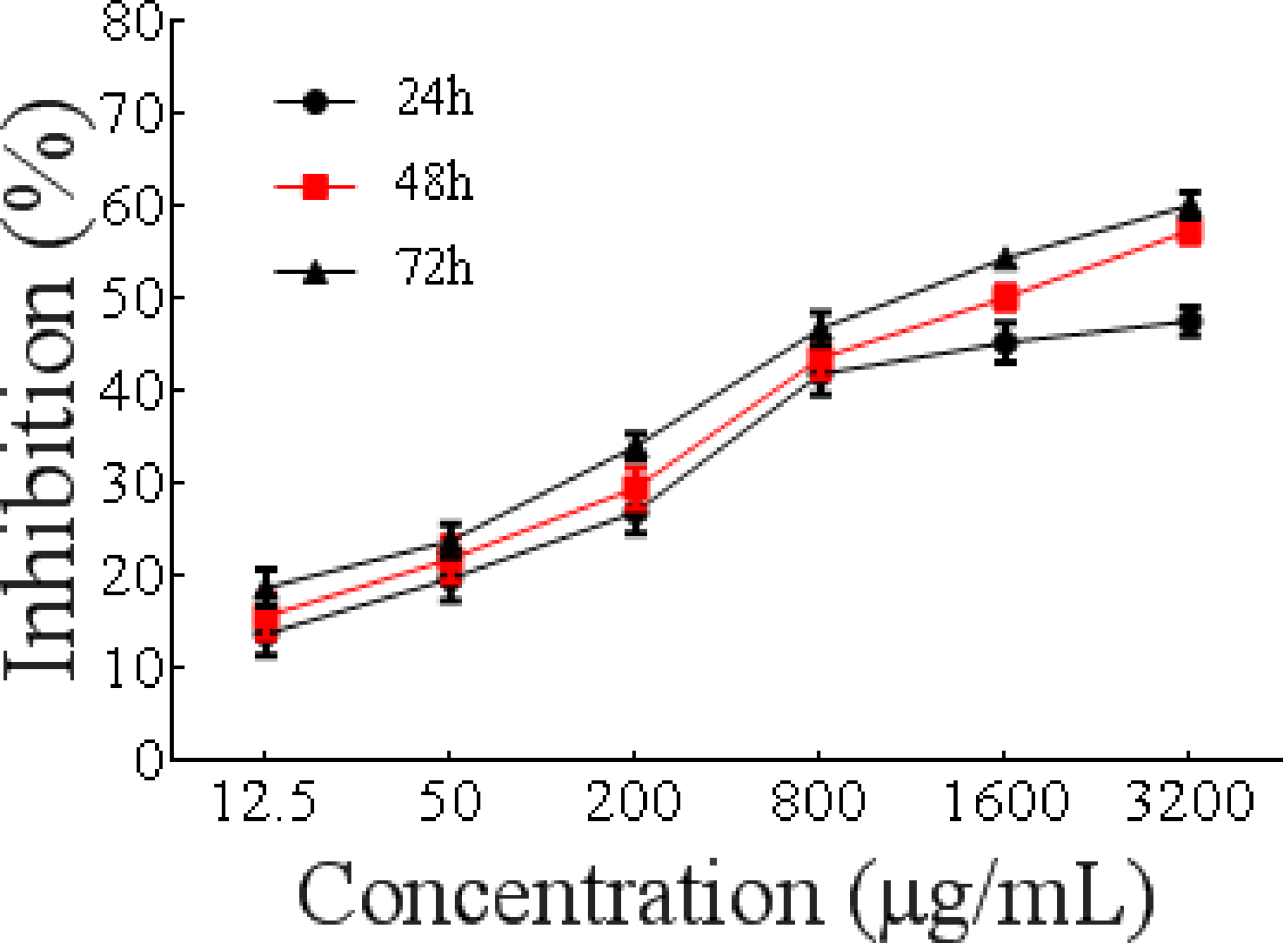
Time curve of MCF-7 cells proliferation as a function of PSO concentration.

As shown in Figure 5, PSO inhibited the growth of MCF-7 cells in a dose- and time-dependent manner with IC_50_ values of 3179 ± 0.02242 μg/mL, 1566 ± 0.01691 μg/mL, and 1064 ± 0.01413 μg/mL, respectively, after 24 h, 48 h and 72 h. PSO significantly inhibited the proliferation of Eca-109 cells.

##### Growth inhibition of vero cells

**Figure 6:**
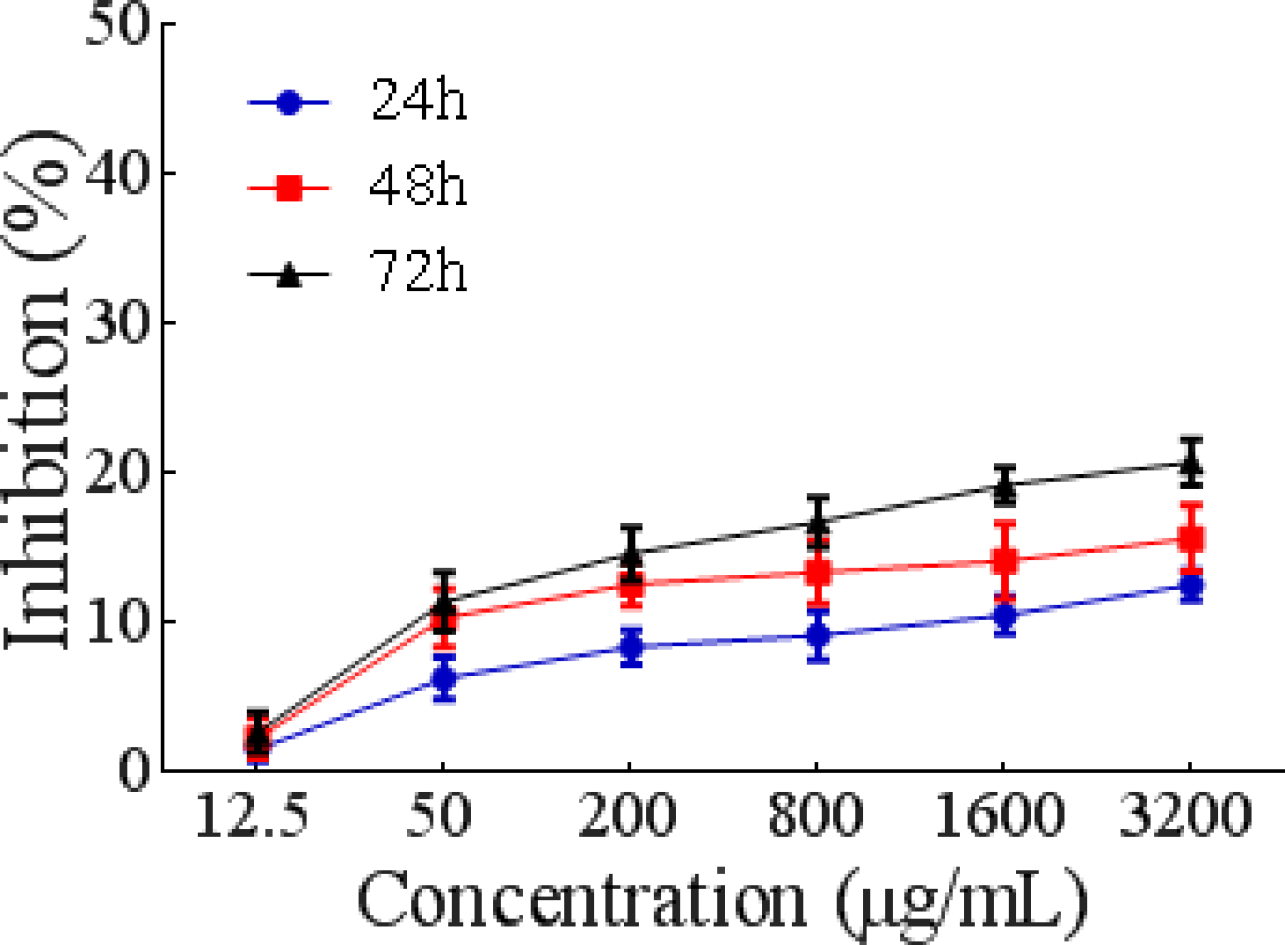
Time curve of vero cells proliferation as a function of different concentrations of PSO.

As shown in Figure 6, the antiproliferative effect of PSO on Vero cells has a slightly increasing trend in a concentration- and time-dependent manner. However, the inhibitory rate of PSO against Vero cells was only 19.25 ± 1.2162% at a concentration of 1600 μg/mL after a 72 h incubation time. The inhibitory rate against MCF-7 cells was up to 54.51 ± 1.1738% (p<0.05), while against HeLa cells, the rate was up to 54.42 ± 1.7466% (p<0.05). For Eca-109 cells, the rate was up to 44.33 ± 2.3405% (p<0.05) under the same conditions. Therefore, our results indicate that PSO had less cytotoxic effect on the normal Vero cells. Overall, the growth inhibition of Vero cells by PSO was much weaker than that of MCF-7, HeLa, and Eca-109 cells.

In summary, the above *in vitro* studies clearly show that the inhibitory effect of PSO on MCF-7 cells was stronger than that on HeLa and Eca-109 cells. This is the first report that demonstrates inhibition of the breast cancer cell growth *in vitro* by PSO. This report suggests a potential therapeutic role of PSO in the treatment of breast cancer. Further research on the mechanism of PSO inhibition of MCF-7 cell proliferation remains to be conducted.

##### Application of PSO to horse oil storage

Due to the adverse health effects of synthetic antioxidants, such as TBHQ and butylated hydroxyanisole (BHA), there has been a considerable increase in demand for isolating naturally occurring bioactive molecules for the food and pharmaceutical industries [35]. In this study, we compared the antioxidant effect of PSO to the synthetic antioxidant TBHQ in horse oil storage. As shown in Figure 7a, the addition of both PSO and TBHQ showed improved protection against auto-oxidation of horse oil. The POVs were much lower than those of horse oil alone, but PSO was weaker than TBHQ in inhibiting lipid peroxidation. This implies that we can further improve horse oil stabilization if PSO and TBHQ are combined, producing a synergistic effect [36] while minimizing the amount of synthetic antioxidant.

Additionally, we investigated the dose-dependent effect of PSO on storage stability of horse oil. We found that the lipid antioxidant capacity of PSO increased with the dose. This is significantly different from the control group, which showed a significant increasing trend the POV in the absence of PSO (Figure 7b). This result suggests that PSO can be used as a natural preservative in the food and cosmetics industries.

## Conclusions

With increased knowledge of the important bioactive molecules in seed oils, such as lipids and pigments, seed oils that possess anti-oxidation and anti-tumor potential have gained significant attention for the treatment of tumors and other cancer-related problems [37,38]. We tested the anti-oxidation and anti-tumor cell proliferation activities of PSO. Our study showed that PSO had remarkable free radical scavenging capabilities, as its hydroxyl free radical scavenging activity is stronger than that of TBHQ. PSO also displayed a stronger antiproliferative effect on MCF-7 cells than on either HeLa or Eca-109 cells with a dual-dependent relationship in a time- and dose-dependent manner. Moreover, PSO protects horse oil against lipid oxidation in a dose-dependent manner. Our findings suggest that PSO can be useful as a health food for the prevention and mitigation hypertensive symptoms and cosmetic ingredient. Studies on the mechanisms of anti-oxidation and anti-tumor cell proliferation are currently underway.

## Figures and Tables

**Figure 1a:**
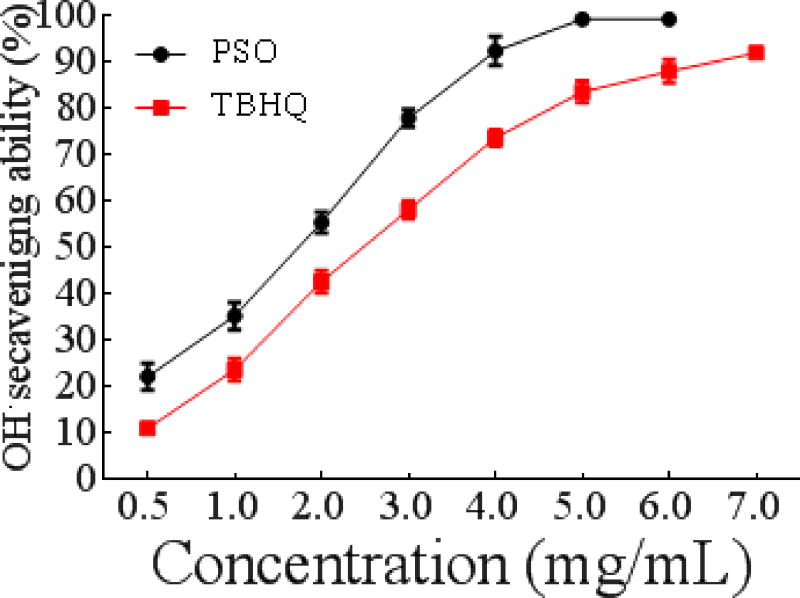
Antioxidant activity of PSO *in vitro*. Hydroxyl radical scavenging ability.

**Figure 1b:**
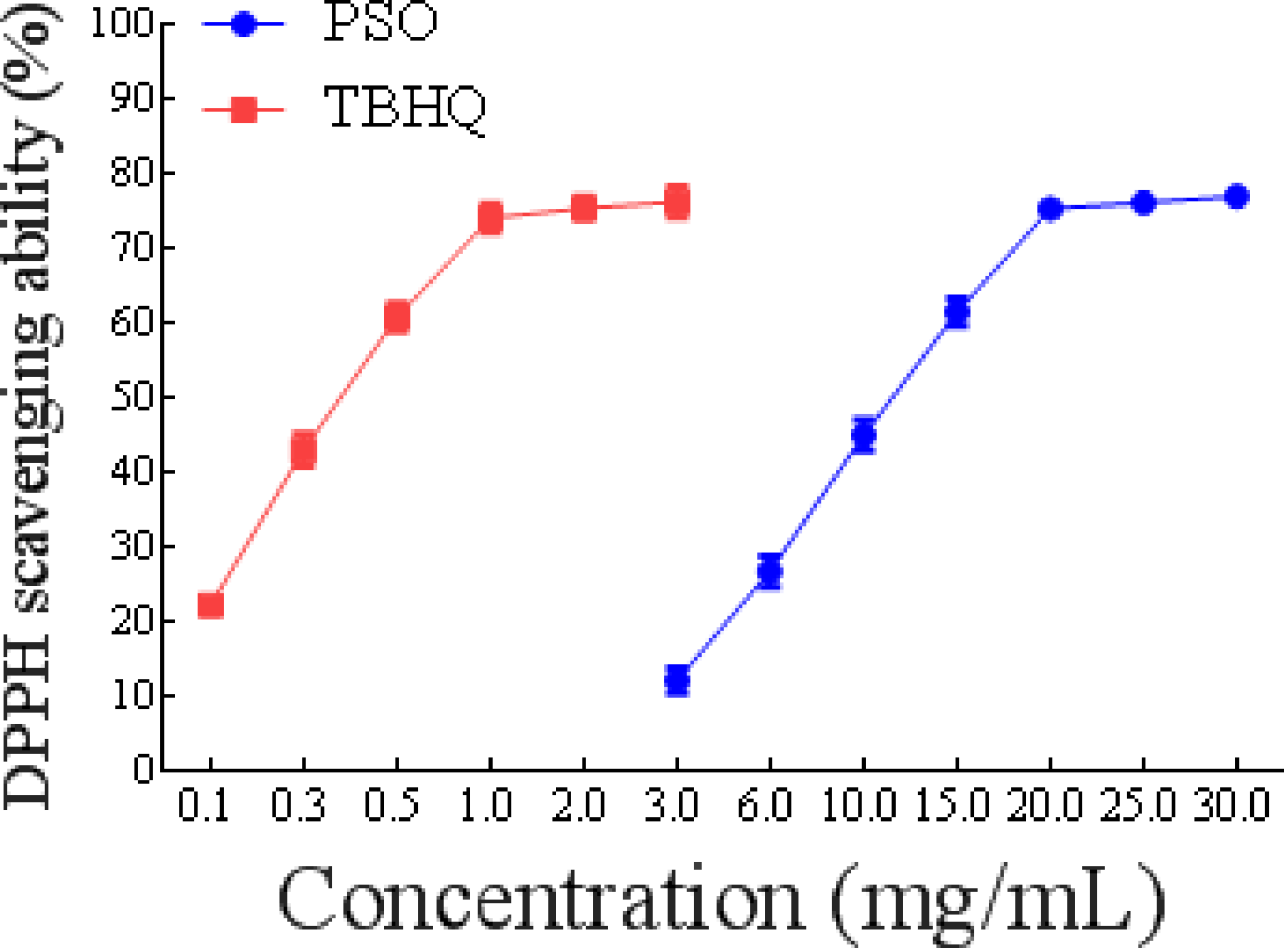
Antioxidant activity of PSO in vitro. DPPH• radicals scavenging ability.

**Figure 1c:**
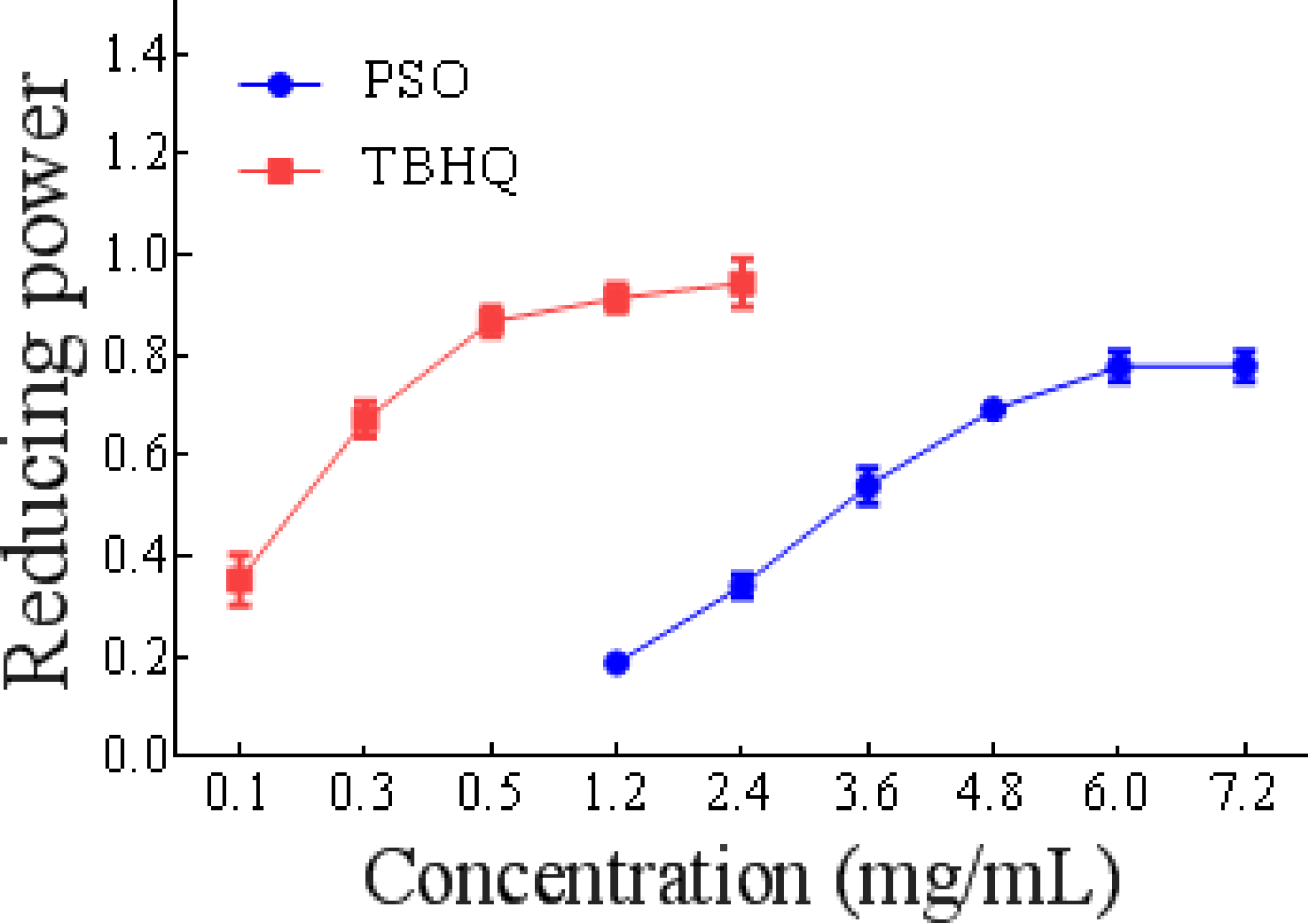
Antioxidant activity of PSO *in vitro* reducing power.

**Figure 7a:**
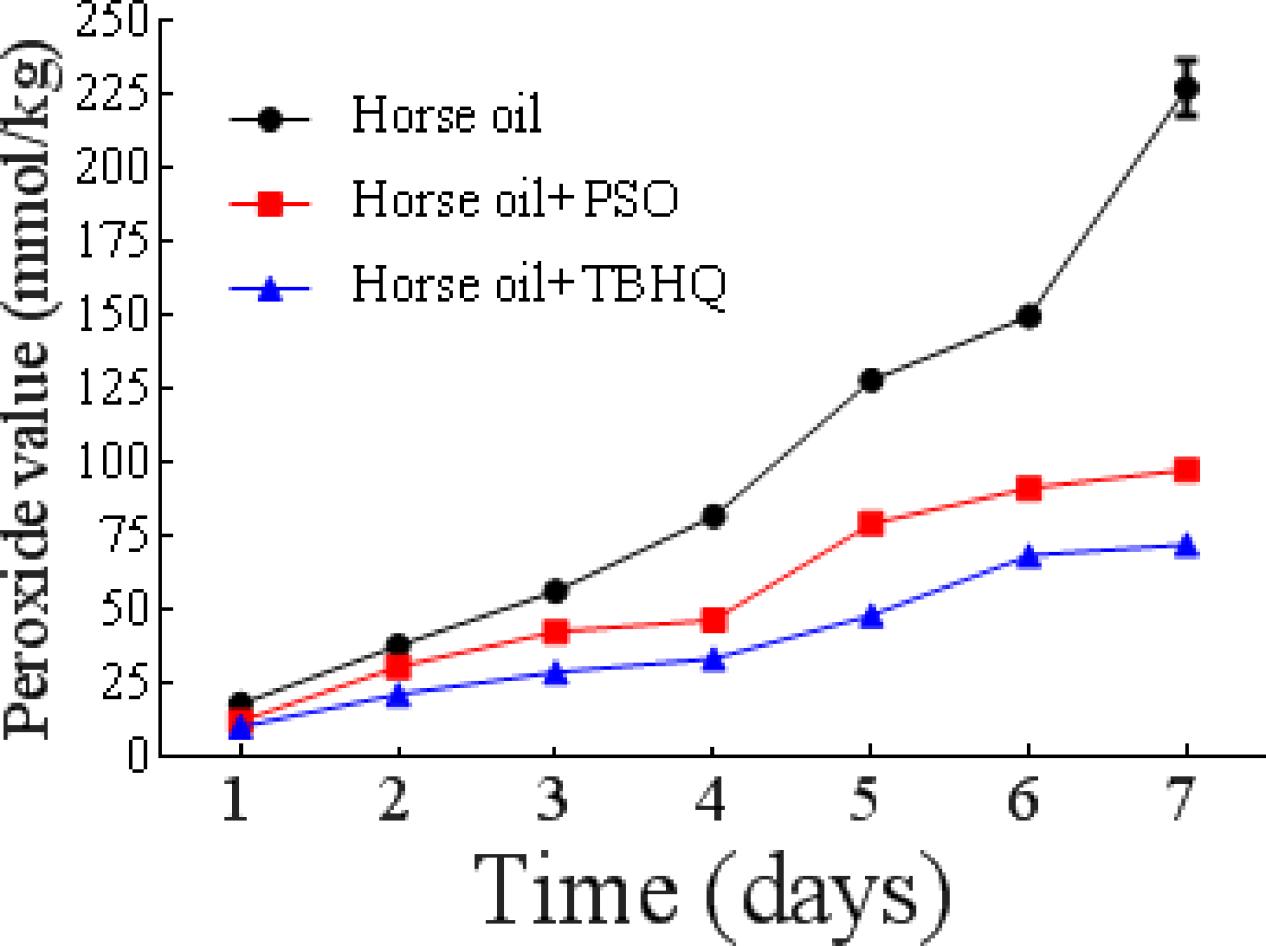
Effect of PSO on the oxidative stability of horse oil during storage. Effect of PSO and other antioxidants on horse oil storage stability.

**Figure 7b:**
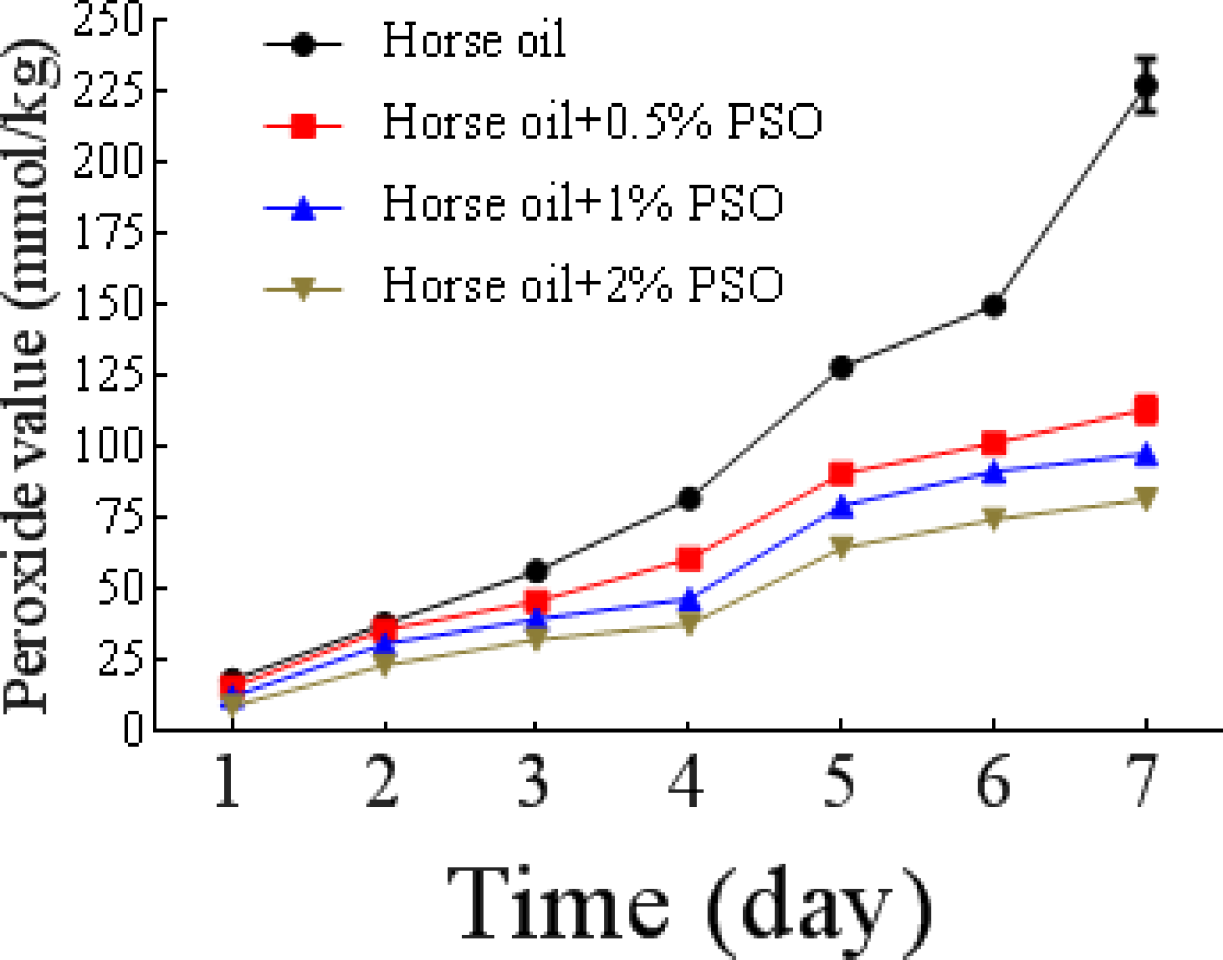
Effect of PSO on the oxidative stability of horse oil during storage. Effect of the PSO dose on storage stability of horse oil.
